# Re-searcher: a system for recurrent detection of homologous protein sequences

**DOI:** 10.1186/1471-2105-9-296

**Published:** 2008-06-27

**Authors:** Valdemaras Repšys, Mindaugas Margelevičius, Česlovas Venclovas

**Affiliations:** 1 Institute of Biotechnology, Graičiūno 8, LT-02241 Vilnius, Lithuania; 2 Faculty of Mathematics and Informatics, Vilnius University, Naugarduko 24, LT-03225 Vilnius, Lithuania

## Abstract

**Background:**

Sequence searches are routinely employed to detect and annotate related proteins. However, a rapid growth of databases necessitates a frequent repetition of sequence searches and subsequent analysis of obtained results. Although there are several automatic systems available for executing periodical sequence searches and reporting results, they all suffer either from a lack of sensitivity, restrictive database choice or limited flexibility in setting up search strategies. Here, a new sequence search and reporting software package designed to address these shortcomings is described.

**Results:**

Re-searcher is an open-source highly configurable system for recurrent detection and reporting of new homologs for the sequence of interest in specified protein sequence databases. Searches are performed using PSI-BLAST at desired time intervals either within NCBI or local databases. In addition to searches against individual databases, the system can perform "PDB-BLAST"-like combined searches, when PSI-BLAST profile generated during search against the first database is used to search the second database. The system supports multiple users enabling each to separately keep track of multiple queries and query-specific results.

**Conclusions:**

Re-searcher features a large number of options enabling automatic periodic detection of both close and distant homologs. At the same time it has a simple and intuitive interface, making the analysis of results even for a large number of queries a straightforward task.

## Background

Protein sequence database searches are routinely employed to detect homologs of the sequence used as a query. However, at present, protein sequence databases are growing exponentially necessitating frequent repetition of searches to find out whether new homologous sequences were added. The analysis of results obtained during such repeated searches may also be tedious and time consuming. The task of manually keeping up with changes in databases becomes unbearable if one is interested in finding new homologs not for a single sequence, but for a few or few dozen sequences. To help cope with the periodic detection of new homologs a number of automatic procedures have been developed including Swiss-Shop [1], DBWatcher [2], BLAST Search Updater [3], ReHAB [4] and DbW [5]. Most of them use BLAST [6], a popular sequence search engine. While BLAST is good in detecting closely related sequences, distant relatives may often escape undetected. Some recent systems for periodic searches use more powerful homology detection tools. For example, ReHAB [4] uses PSI-BLAST and DbW [5] utilizes a collection of several methods. ReHAB is designed to handle a large number of query sequences, while DbW attempts to include only functionally related new sequences. Both are very efficient in performing their tasks but their common caveat is that most parameters for the searches are predefined and users are left with the choice "love it or leave it".

Re-searcher is a new software package that is designed to circumvent these caveats and provide the user with a highly configurable environment for performing recurrent sequence searches. Because of the flexibility in setting up sequence search strategy and specific parameters Re-searcher can be used to find both closely related family-specific homologs and very distantly related matches.

## Implementation

**Figure 1:**
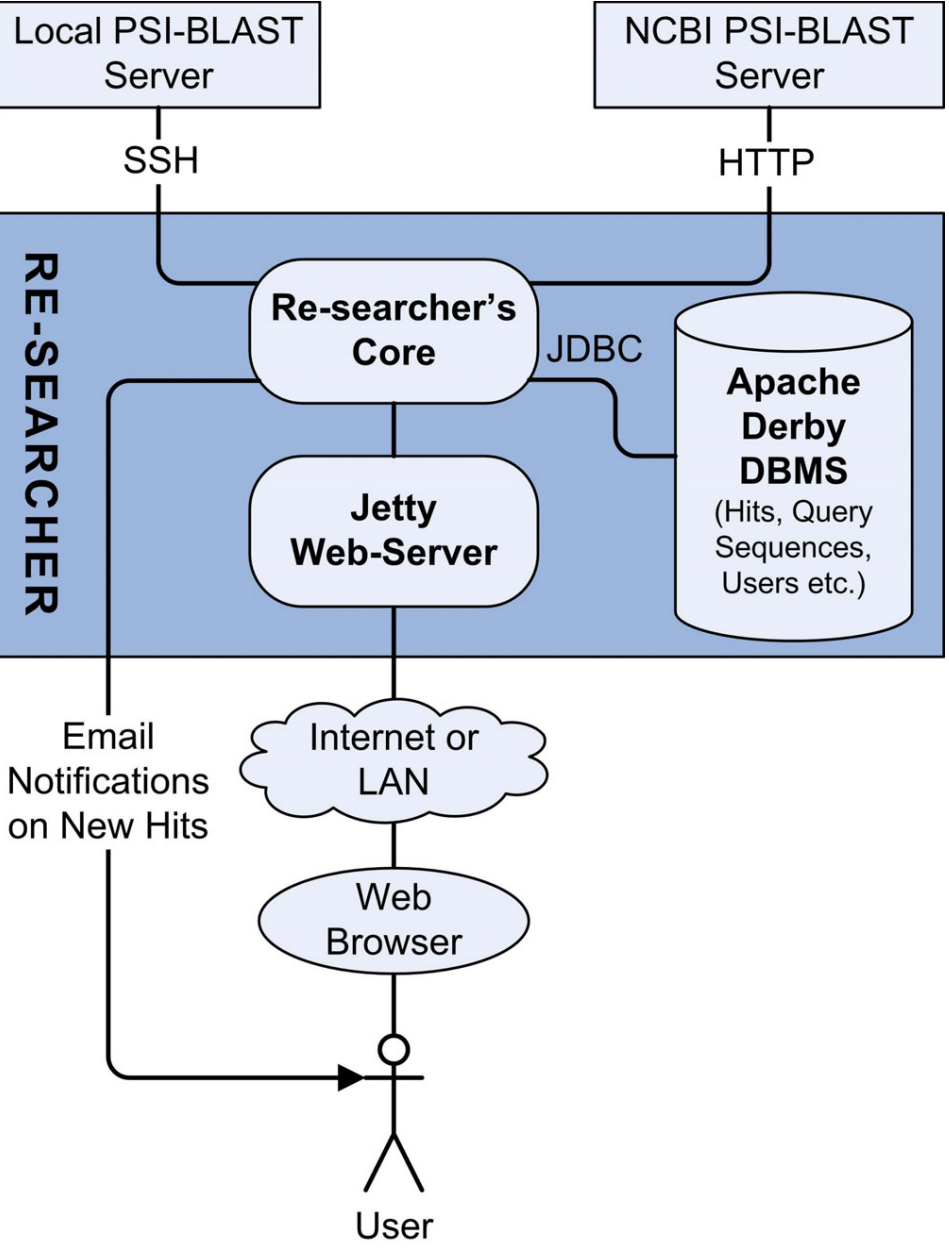
**Organization of the Re-searcher system**. Users interact with Re-searcher through a web browser. HTTP requests from the web browser are handled by the integrated web server. Re-searcher uses a relational database to store all the data (queries, hits etc.). Sequence searches are performed either remotely (at NCBI) or locally.

Re-searcher is written in Java thus making it easily portable to different operating systems (with Sun's Java Runtime Environment Version 6.0 or later installed). It has been tested on Linux and Windows computers. The Re-searcher system includes several components (Fig. 1). The user interface is provided through the web browser. Sequence search data are handled by the Apache Derby database management system [7]. Re-searcher has the Jetty [8] web server embedded, but it is also possible to run the application on any other Java web server. "Out-of-the-box" installation provides all the basic components that enable user to perform remote searches at NCBI. For local searches Re-searcher must be configured to work with locally installed BLAST suite of programs and sequence databases. The system is designed such that Re-searcher and the local BLAST server don't have to reside on the same computer, only the SSH connection between them should be enabled. The user can communicate with Re-searcher either from a local computer or remotely through the internet. Re-searcher has an option to inform the user of new matches by e-mail. For this option to be functional an SMTP mail server must be accessible. All these configuration parameters can be set up through the administrator's account.

## Results

### Overview of the system

To detect new homologous protein sequences Re-searcher uses PSI-BLAST as the search engine. However, if only BLAST functionality is desired, Re-searcher can be configured to run only a single PSI-BLAST iteration. Searches can be performed at specified time intervals against either NCBI [9] protein databases or locally installed custom sequence databases. The user is able to individually configure both the search parameters and the search periodicity for each query. Once query is entered into the system Re-searcher performs sequence search automatically using specified parameters at every query-specific time interval. During every search all the detected sequences are compared to those, found for the query during all previous searches, and only non-identical sequences are added to the Re-searcher's database and reported as new.

Re-searcher provides a possibility to do more than just straightforward recurrent PSI-BLAST searches. It can perform combined searches involving two databases. Such strategy is useful if the user is interested in detecting remote homologs within a small sequence database. The direct searches against such database may be unable to generate rich sequence profiles that are the main strength of PSI-BLAST. Therefore, Re-searcher provides a possibility to run an iterative search against the first (large) sequence database and then use the obtained profile (Position Specific Scoring Matrix or PSSM) to search against the smaller second, either a specialized or private, database. An example of such scheme is so-called "PDB-BLAST", when the generated PSI-BLAST profile is used to detect distantly related sequences that have known PDB structures.

In addition to the familiar NCBI-style PSI-BLAST form for setting up individual queries, Re-searcher provides informative easy-to-understand reports. Queries, for which new homologs have been detected, as well as newly detected matches within the list of all homologs found so far, are clearly marked. To simplify the analysis, resulting lists of homologs can also be sorted and filtered.

Re-searcher supports multiple users. Each user can have an individual account, which is not visible to the public and holds all the user-specific queries and results.

### Setting up queries

**Figure 2:**
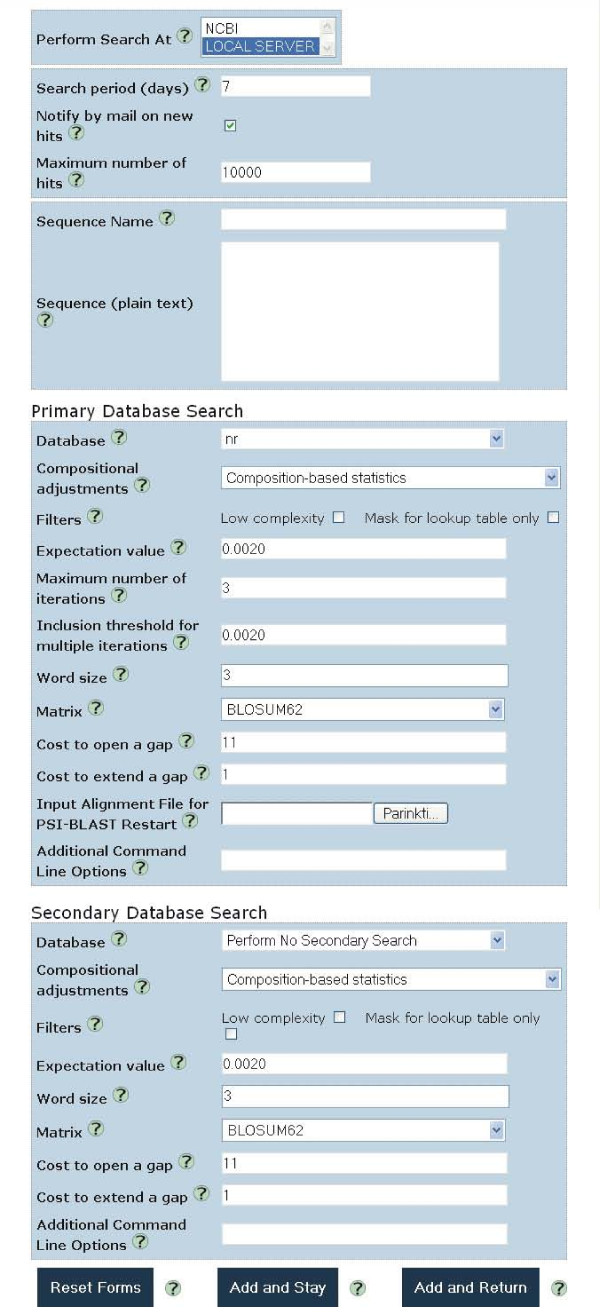
**Query input form**. The form consists of three parts: the top part is for setting general parameters and query sequence input, the middle part is for setting PSI-BLAST search parameters in the first database, and the bottom part is for setting the search in the second database. By default a search only against the first database is performed, but if the second database is also selected then both databases are used in a combined search.

Each query is set individually allowing for maximum flexibility in defining the search strategy and reporting. The query input form is designed such as to provide all the essential options available on the PSI-BLAST web page at NCBI (Fig. 2). Searches for a query can be set up to be performed either locally or at NCBI. Most of the available search options are independent of the chosen location, yet local searches offer some distinct advantages. For example, the user can make the detection of new homologs more sensitive and more specific by providing a curated multiple alignment as an input into the PSI-BLAST search. Another difference is that remote searches can only utilize databases that are available from NCBI while there is no such limitation for local searches.

In addition to straightforward searches against the specified database, both local and remote setups offer a combined two-database searching discussed above. Although it is possible to do the same kind of a combined search through the PSI-BLAST web page at NCBI, it can only be done manually in a number of steps.

The setup for a new query also includes the search periodicity parameter and an option to notify the user of newly detected matches by e-mail.

### Reporting of the new matching sequences ("hits")

One of the common ways to inform a user of new matches to the query is to send an e-mail notification. However, sending detailed information about all new hits is not necessarily a good idea. For example, the initial search using multiple PSI-BLAST iterations can sometimes generate an overwhelming number of hits. If all this information is e-mailed to the user, the mailbox might easily get clogged.

**Figure 3:**
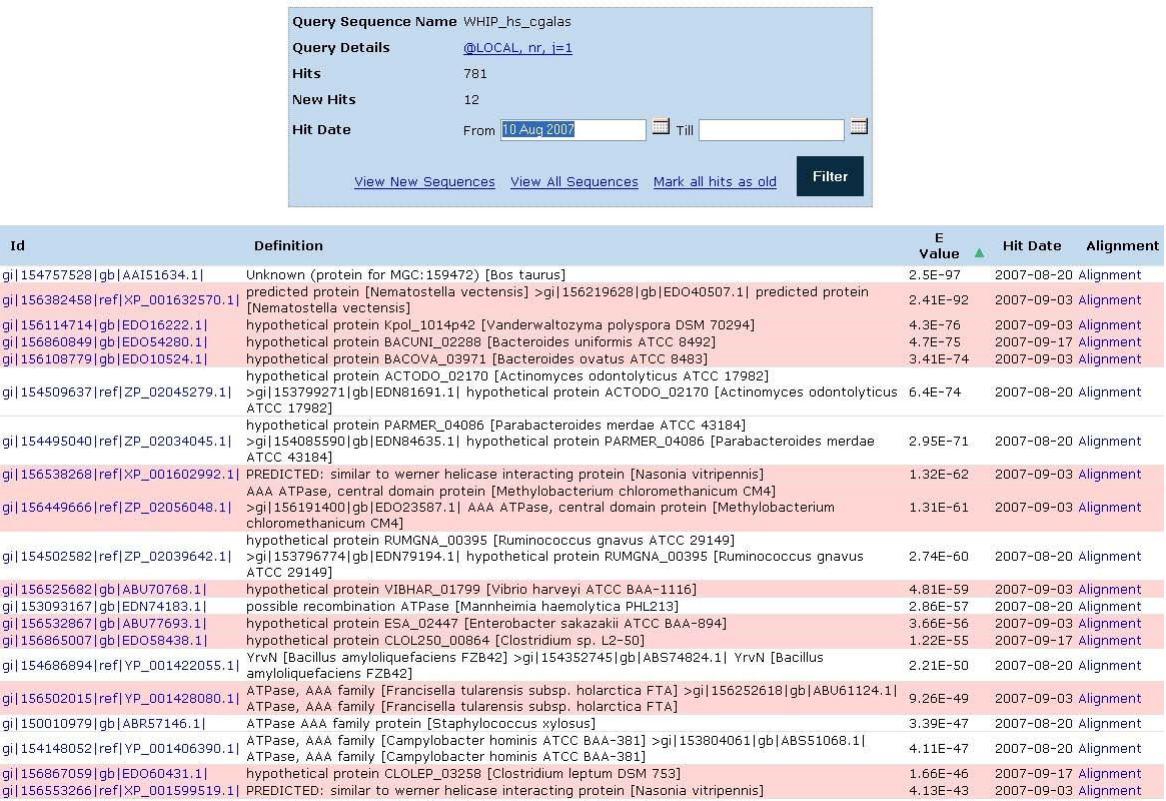
**Query-specific results report**. Summary of the results including sequence name, a link to the query-specific search parameters, both the total number and the number of new hits is provided at the top. Below, the results table by default provides data on all accumulated hits, but the user can choose to display only hits found within certain dates. The results table provides a description of each hit, PSI-BLAST E-value and the date the hit was detected. The table can be sorted by any of these attributes. Links within the "Id" column lead to the sequences of the hits; the "Alignment" links lead to the actual alignments between the query and the corresponding hits. If hits have associated GenInfo (GI) numbers then they are also linked to Entrez reports at NCBI. Newly detected hits remain highlighted until the user changes their status by pressing "Mark all hits as old".

Re-searcher uses a more efficient way to report of newly detected hits. It sends a very short e-mail report, which contains an embedded link to the query-specific results page. The results page for each query reports a short summary including parameters of the search and a complete list of detected sequences (Fig. 3). For each of them a description contained in the FASTA header, statistical significance (E-value), the detection date and links to both the sequence and the corresponding alignment are displayed. New matches are colored differently for easy identification. They remain new until the user explicitly sets their status as old. Also, either all or only newly detected sequences can be displayed and saved in multiple FASTA format. To simplify the analysis of the results, hit lists can be sorted by the inclusion date, E-values or the description text. In addition, hit lists can be filtered to include only those sequences that were detected within specified dates. This feature is very useful if over time hits grow to a very large number and the analysis of the entire list becomes impractical.

**Figure 4:**
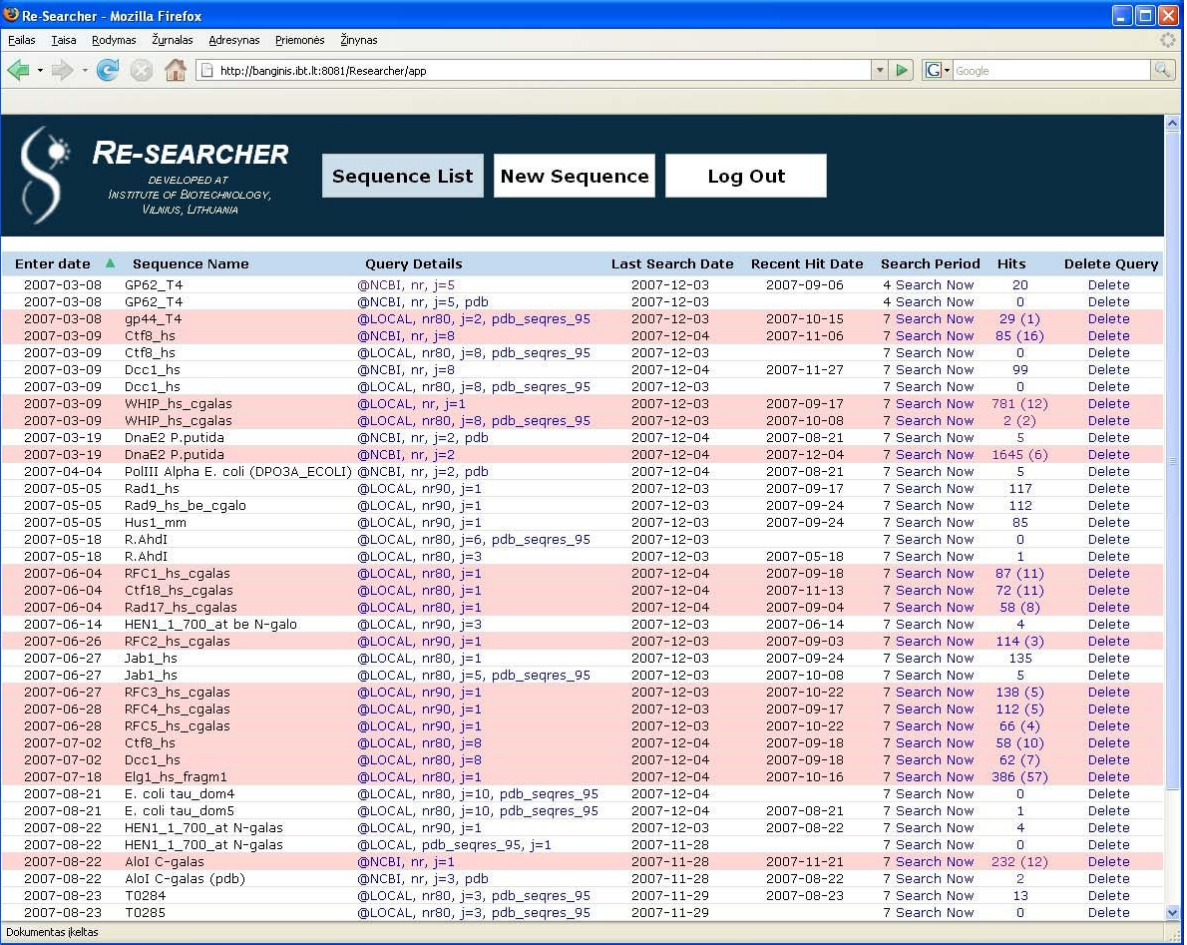
**User's workspace: the top layer**. The top layer of the user's workspace provides "Sequence List" – a table of short summaries for each query. Queries, for which there are new matches, are highlighted. The summary table can be sorted by any column. Links within the "Hits" column lead to complete query-specific results, while "Query Details" link to complete list of parameters associated with the search. By pressing "Delete" the entry is removed. The "Search now" link provides a possibility to initiate search immediately without waiting for a scheduled run. New query can be added using the "New Sequence" menu item at the top.

The results can also be accessed independently of e-mail notifications by directly logging into Re-searcher through the user's account. In this case, query-specific results are accessed through the user's workspace at the top level (the main page), which displays the list of all the queries, each one having a brief but informative single-line status report (Fig. 4). This report tells the user what was the search strategy, how many total hits were found and how many of them are new. It also tells the search periodicity and dates when the query was entered, when it was last searched, and when was the last time new hits were found. The queries that have new hits are easy to see since in the list they appear with a different color. In addition, the list of queries can be sorted by any of the attributes (columns) enabling the user to quickly get an overview even for a large number of queries. The main page is the place where the user can add or delete queries. It also enables the user to perform search for any query immediately, without waiting for the scheduled run.

### User management

Re-searcher is designed to support multiple password-protected user accounts. Depending on the desired user policy, the system may be configured to either allow unhindered creation of new user accounts or have the administrator be in charge of addition of new users. The administrator's account is also used to set up general parameters for the system such as the IP address of the local BLAST server, paths to the suite of BLAST programs and local databases. Of course, it is perfectly possible to use Re-searcher in a single-user mode.

## Conclusions

The Re-searcher system is designed to answer a growing need among both computational and experimental biologists to be kept updated on a regular basis about new homologs for the protein sequence(s) of interest. Re-searcher combines the simplicity of installation and use with the flexibility of setting up sequence searches according to the needs of the user. More specifically, the system allows individualized search and reporting strategies for each query, including the search periodicity, choice of databases (remote or local), single- or two-database searches and various search parameters. Re-searcher can be run both in a single-user mode (e.g. on a PC) and as a centrally managed service for multiple users.

## Availability and Requirements

**Project name**: Re-searcher

**Project home page**: ; 

**Operating system(s)**: Platform independent

**Programming language**: Java

**Other requirements**: Sun's Java Runtime Environment Version 6.0 or later

**License**: GNU GPL

**Any restrictions to use by non-academics**: No restrictions

## Authors' contributions

VR designed and developed the Re-searcher system. MM participated in the design and testing of the software. ČV conceived of the project, coordinated the software development, tested the system and drafted the manuscript. All authors read and approved the final manuscript.

## References

[B1] Swiss-Shop. http://www.expasy.org/swiss-shop/.

[B2] DBWatcher. ftp://ftp-igbmc.u-strasbg.fr/pub/DBWatcher/.

[B3] Boone M, Upton C (2000). BLAST Search Updater: a notification system for new database matches. Bioinformatics.

[B4] Whitney J, Esteban DJ, Upton C (2005). Recent Hits Acquired by BLAST (ReHAB): a tool to identify new hits in sequence similarity searches. BMC Bioinformatics.

[B5] Prigent V, Thierry JC, Poch O, Plewniak F (2005). DbW: automatic update of a functional family-specific multiple alignment. Bioinformatics.

[B6] Altschul SF, Madden TL, Schaffer AA, Zhang J, Zhang Z, Miller W, Lipman DJ (1997). Gapped BLAST and PSI-BLAST: a new generation of protein database search programs. Nucleic Acids Res.

[B7] Apache Derby. http://db.apache.org/derby/.

[B8] Jetty web server. http://www.mortbay.org/.

[B9] NCBI. http://www.ncbi.nlm.nih.gov/.

